# Long-term outcome of endometrial ablation therapy with Cavaterm Thermal Balloon in patients with abnormal uterine bleeding

**DOI:** 10.4274/jtgga.galenos.2019.2019.0107

**Published:** 2020-03-06

**Authors:** Mojgan Karimi-Zarchi, Marzieh Fathi, Afsar Tabatabaie, Farimah Shamsi, Leila Allahqoli, Leila Zanbagh, Seyed Mohammad Amin Hashemipour, Liselotte Mettler

**Affiliations:** 1 Endometriosis Research Center, Iran University of Medical Sciences, Tehran, Iran; 2 Department of Obstetrics and Gynecology, Shahid Sadoughi University of Medical Sciences, Yazd, Iran; 3 General Practitioner Medicine, Shahid Sadoughi University of Medical Sciences, Yazd, Iran; 4 Department of Epidemiology, Shahid Sadoughi University of Medical Sciences, Yazd, Iran; 5 Department of Medical Teaching, Islamic Azad University Faculty of Medicine, Young Researchers and Elites Club, Yazd, Iran; 6 Department of Obstetrics and Gynecology, Schleswig-Holstein University Hospital, Kiel, Germany

**Keywords:** Abnormal uterine bleeding, endometrial ablation, hysterectomy, amenorrhea

## Abstract

**Objective:**

The purpose of this study was to evaluate the long-term outcome of endometrial ablation (EA) therapy with Cavaterm Thermal Balloon in patients with abnormal uterine bleeding (AUB).

**Material and Methods:**

The retrospective cross-sectional study was performed on 209 patients who referred to Shahid Sadoughi Hospital in Yazd, Iran between March 2010 and September 2017 with AUB undergoing EA therapy. The data was collected by a questionnaire from the medical records of patients and phone call. The primary and secondary outcomes post EA therapy (from six months to seven years post-operatively) were assessed in patients.

**Results:**

The mean age of participants was 45.9±5.9 years and the mean follow-up duration was 21.2±13.2 months. The rate of treatment response was 95% in the first six months and 92.1% thereafter. The prevalence of amenorrhea was 41.2%. The patient satisfaction rate at the end of follow-up duration was 81.3%. Dysmenorrhea completely resolved in 32.6%. Moreover, 1.4% of patients became pregnant during follow-up. By the end of follow-up, four (1.9%) patients had a hysterectomy due directly to treatment failure.

**Conclusion:**

This study showed that EA surgery with Cavaterm Thermal Balloon was an effective treatment for AUB. The procedure was safe and was associated with a very low rate of postoperative adverse events. The patient satisfaction rate was favorable.

## Introduction

Abnormal uterine bleeding (AUB), which refers to any irregularity in the menstrual cycle, is one of the most common causes of women of childbearing age being referred to clinics (1). Approximately 16% of hysterectomies occur due to AUB (2). Hysterectomy is a definitive treatment for AUB and has been reported to be the second most common major surgical procedures in the United States (3). A strong preference for preservation of the uterus in developed countries has recently led to greater use of minimally invasive drug therapies, including Mirena intra-uterine device (also known as the levonorgestrel releasing device or LNG-IUS) and endometrial ablation (EA), even in cases where there is no desire for future pregnancy (4,5). There is also a contraindication for drug therapies in cases with co-existing diseases such as diabetes and cardiovascular disease (6). EA therapy is preferred to hysterectomy due to the benefits of being outpatient based, being quicker, with fewer complications, any hospital stay is usually shorter and recovery is faster too (7). EA is performed using two methods: hysteroscopic endometrial ablation (HEA); and non-HEA (NHEA). HEA uses laser, electric current, or heat energy for coagulation or evaporation of the tissue. The NHEA approach is performed using EA computer systems with the aid of electric current, hyperthermia, cryotherapy or microwaves (8). The purpose of this study was to evaluate the long-term outcomes of EA therapy with the Cavaterm Thermal Balloon in patients with AUB.

## Material and Methods

This was a retrospective cross-sectional study which was performed at Shahid Sadoughi Hospital in Yazd, Iran in 2018. All aspects of this research were approved by Ethics Committee of the Yazd Shahid Sahoughi University of Medical Sciences (IR.SSU.MEDICINE.REC.1396.186).

In this study, we reviewed medical record of 256 patients who had undergone EA between March 2010 and September 2017. These patients had been referred to Shahid Sadoughi Hospital with AUB, who did not respond to drug therapies or had an impediment to drug and surgical treatment or were reluctant to perform hysterectomy. All participants had completed informed consent before surgery.

Enrollment criteria were: 1) premenopausal women ≥18 years old; 2) unwillingness to maintain fertility and no desire for pregnancy; 3) no urogenital infection; 4) natural history of cervical cytology; 5) negative Beta human choronionic gonadotropin test; 6) no contraindication for EA surgery; 7) underwent EA (Cavaterm Thermal Balloon) after March 2010; and 8) had documented follow-up ≥6 months.

### Ablation procedure and follow-up

Vaginal ultrasound was performed before surgery and the thickness of the myometrium, uterine cavity length and myometric length were measured. Endometrial curettage was then carried out to reduce endometrial thickness and the samples were sent for pathological examination. After placing an anesthetic mask, the patient was placed in a lithotomy position. The lower abdominal region, vulva, femoral region, and vaginal cavity were sterilized with iodine. The cervix was initially opened using a 6 mm dilator, followed by using a cavaterm system comprising a silicon balloon connected to a catheter with a width of 6 mm and a unit (thermal balloon EA device and catheter, Plus cavaterm TM model, (Veldana Medical SA Co., Switzerland). The silicone balloon length was set based on the measurements of each individual uterine cavity. After emptying the air from the cavaterm system, the catheter end was inserted into the fundus, and the balloon was filled by glucose 5% fluid until the pressure reached 230±10 mmHg, and this pressure was maintained until the end of the treatment. Then, the circulation of fluid and heat was begun. EA started after reaching a temperature of 70±10 °C. The treatment was continued at this temperature for 10 minutes and then the heating was stopped, the fluid was pumped out and the catheter was removed. For removal the EA catheter was surrounded by an insulator to prevent thermal damage of the cervix and vaginal canal. The patient was then transferred to the recovery ward.

Patients were followed up for six to 90 months after EA therapy. In this study follow up period of patients was divided into four periods of up to six months, six to 12 months, twelve to 24 months and more than 24 months after surgery.

### Outcome measures

The primary outcomes were changes in duration and interval of menstruation, amenorrhea rate, and bleeding reduction of at least 50% after surgery. It should be noted that amenorrhea rate and bleeding reduction of at least 50% six months after surgery were considered as the criterion for treatment response.

The secondary outcomes were the prevalence of anemia, dysmenorrhea, patient satisfaction, secondary intervention (medical or surgical) for recalcitrant AUB, adverse effect of EA therapy, and comparison variables in two groups of treatment respond and treatment failed. Anemia was defined if hemoglobin levels were lower than 12 mg/dL (9). Dysmenorrhea had been recorded using a 10-point visual analog scale in the medical record of patients, which higher points of three being considered as a dysmenorrhea (10), adverse effect of EA therapy including of blood discharge, fever (defined as body temperature of >37.5 °C), extreme and prolonged suprapubic pain, urinary tract infection, vaginosis, malodorous discharge, vomiting, and uterine rupture.

The primary and secondary outcomes of post EA therapy (from six months to seven years post- operatively) were assessed in patients.

Data collection was performed by means of a questionnaire and data was extracted from the medical records of the patients, telephone consultation with the patients.

### Statistical analysis

The collected data were entered in the statistical software program IBM SPSS Statistics for Windows version 20.0 (SPSS Inc., Chicago, IL, USA). Descriptive statistics (mean ± standard deviation, frequency, and percent) were used to present the data. Categorical variables were assessed with chi-squared and Fisher’s exact test. Continuous variables were compared by Student’s t-test. For all tests, p-values <0.05 indicated statistically significant differences.

## Results

Of 256 existing medical records of patients with EA between March 2010 and September 2017, one patient was omitted due to hysterectomy during an initial examination. The reason of her hysterectomy was suspicion of endometrial cancer which proved to be metastatic sarcoma and was treated with radiotherapy after surgery. A further 17 patients had not attended a postoperative follow-up. Ten patients did not answer the phone call, and nineteen patients did not accept to participate in the study. Ultimately, the analysis was performed with the data of 209 patients.

The mean age of the patients was 45.94±5.9 years. The reasons for undergoing EA were: desire to preserve the uterus and ovaries, and age conditions in 153 patients (73.2%); and the presence of underlying disease as an obstacle to more invasive surgery, such as hysterectomy, in 56 patients (26.8%). All patients had a chief complaint of excessive menstruation and a history of drug treatment. Most patients (75.1%) had normal (proliferative or secretory) pathological results. Patient characteristics pre-EA surgery are presented in Table 1.

The result of primary outcomes in patients before and after EA surgery are presented in Table 2. The mean duration of menstruation was significantly decreased to 3.7±4.3 days in the first six months (p=0.001) and 3.1±3.3 days 24 months after EA surgery (p<0.001). The mean interval of menstruation cycle was significantly increased to 38.5±32.6 days 24 months after EA surgery (p=0.003).

**Figure 1:**
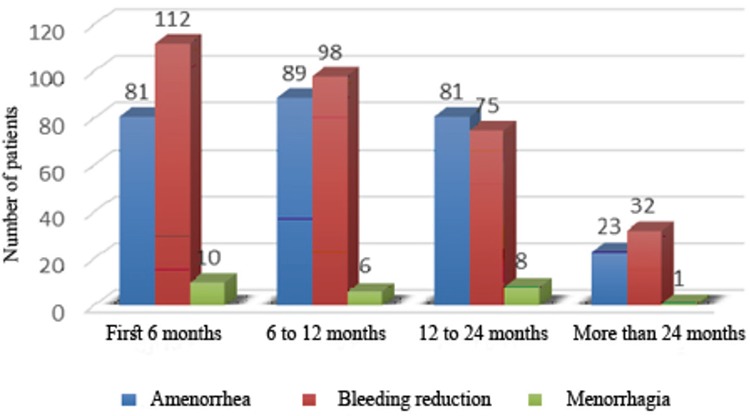
Bleeding state of patients after endometrial ablation therapy

Amenorrhea and bleeding reduction occurred in 193 (95%) in the first six months and in 187 (92.1%) after the first six months. At the end of follow-up, 84 (41.2%) had amenorrhea (Figure 1).

Preoperatively, 146 (69.9%) patients had anemia before surgery and this proportion was significantly reduced after surgery to 61 (29.2%) patients (p=0.001). Of 89 (44.1% of the whole cohort) women who initially experienced dysmenorrhea, only 24 (11.5%) reported that their symptoms had not changed or had worsened, a reduction of 32.6% (Table 3) while the other 65 women reported that their symptoms were “much improved” or “somewhat improved”. A comparison of anemia and dysmenorrhea in patients before and after EA is shown in Table 3.

When patients were queried about overall satisfaction with the EA treatment 89.2% of them reported being either “very satisfied” or “satisfied” versus feeling “neutral” or expressed any degree of “dissatisfaction” (Table 4).

Following EA surgery 62 (29.7%) patients had received secondary intervention for recalcitrant AUB until follow-up. Of that number 38 patients (18.8%) required drug therapy, of which 29 responded (76.3%), mostly to 20 or 40 mg megestrol acetate per day. In addition, 24 (11.5%) patients underwent hysterectomy following EA surgery, 23 of these were in the first three years after the EA procedure.

The most common adverse events after the surgery were blood discharge of more than 14 days in 182 (90.6%) patients. Other adverse events included vaginosis, malodorous discharge, uterine rupture, extreme and prolonged suprapubic pain. The results of patient satisfaction, secondary intervention and adverse events after EA surgery are presented inTable 4.

**Figure 2:**
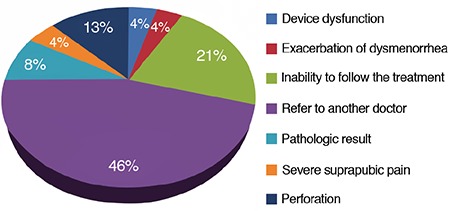
Causes of hysterectomy in patients

Up to the end of the follow-up period, four (1.9%) patients were treated by hysterectomy due to direct result of treatment failure [uterine perforation (n=3), device dysfunction (n=1)] (Figure 2).

The mean age of patients in the treatment failure group was significantly higher than in the treatment response group (49.7 vs 41.2 years; p=0.006). In addition the uterus size (p<0.001) tended to be significantly larger in the treatment failure group. There was no significant relationship between body mass index, gravidity, parity, intrauterine pressure and intrauterine temperature, and result of pathology with treatment failure. The results of comparison of variables in the treatment response and failure groups are presented in Table 5.

**Figure 3:**
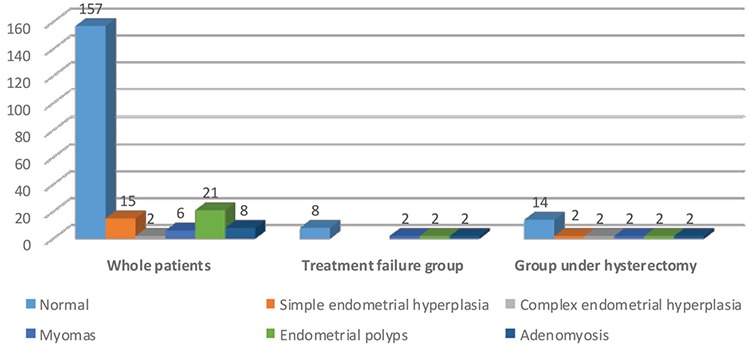
Results of pathology for patients

The pathology result after surgery was reported to be normal endometrium (secretory or proliferative) in 157 patients (75.1%). There was no significant relationship between the pathology type and the treatment response nor was there a significant relationship between the pathology type and the risk of future hysterectomy (p=0.084) (Figure 3).

It is noteworthy that three (1.4%) patients became pregnant in the follow-up period.

## Discussion

In this retrospective study, the outcomes of EA therapy using Thermal Balloon and Plus CavatermTM technique were evaluated in 209 patients with AUB. Study results indicated duration of menstruation, a primary outcome, decreased significantly after treatment and the interval between menstrual cycles also increased significantly. These results are consistent with those of Asgari et al. (11) who reported the duration and intervals of menstruation after EA was significantly decreased and increased respectively. In the Famuyide (12) study the menstrual bleeding rate in the patients with AUB treated with an EA method was reduced, which was associated with lower risk of hysterectomy in the future. In the present study, the rate of amenorrhea was 41.2% at the end of follow-up, which falls into the previously reported rate for amenorrhea, between 19.4 and 58%, in studies of patients with AUB treated with an EA method (11,13,14,15,16,17,18).

In this study, the rates of treatment responses ≤6 and >6 months were 95% and 92.1% respectively which are higher than that in the study of Sharma et al. (19) of 80% and 76% for the first six months and later, respectively. While some studies support the therapeutic role of EA (13,14,15,16,19), unfortunately in some studies recurrent vaginal bleeding had occurred immediately or years after EA surgery (20,21,22). Although the recurrence of vaginal bleeding following EA is attributed to inadequate destruction of the endometrium (20,21), unsuspected deep adenomyosis (22), and development of benign (myomas), or malignant diseases (endometrial hyperplasia, or cancer) may be responsible (23). Therefore, it is suggested that, despite EA rapid treatment response, patients need long follow-ups after surgery due to the risk of bleeding recurrence.

Most of the patients presented in this cohort were anemic before the EA surgery. Bernardi et al. (24) found that a significant percentage of women who report heavy menstrual bleeding are not only iron deficient, but also anemic, although most of their patients with anemia resolved after EA surgery. This was thought to be due to the high rates of amenorrhea and significant bleeding reduction as a result of EA (25). Kim et al. (26) suggested that EA is an effective alternative to hysterectomy for women with persistent menorrhagia and anemia when supportive measures fail.

Dysmenorrhea, defined as a complaint of pain experienced during or immediately before menstruation, improved in the majority of our patients after EA surgery, which is consistent with previous studies (11,17,27). Prostaglandins (PG) and arachidonic acid metabolites play an important role in the pathogenesis of dysmenorrhea, being elevated in women with dysmenorrhea (28). However PGs, together with endothelin, which are powerful, vasoactive substances play a key role in the control of menstrual blood loss (28). Cameron et al. (29) showed the concentration of Prostaglandin E (PGE) and “total” PGs, defined by these authors as PGE + 6oxo PGF1 alpha + PGF2 alpha, was greater in the endometrium of those women with heavy menses than in those individuals with a normal menstrual loss. Therefore, it may be expected that dysmenorrhea will be improved by reducing menstrual bleeding.

The rate of patient satisfaction with treatment was high (86.6%) in our study, consistent with other studies (1,11,12,13,14,18,30). It has previously been reported that the resulting reduction in blood loss and increase in patient satisfaction rates leads to improved quality of life (30,31).

In the present study, following EA surgery 24 (11.5%) patients had subsequent hysterectomy. This is similar to the rates of hysterectomy subsequent to EA therapy, from 10% to 13%, which have been reported previously (16,17,30,32,33,34) although higher rates (18%-25%) have been reported by some studies (30,34). This variability in reported hysterectomy rates may be due to differences in study population and method or technique applied for EA therapy. For example Comino and Torrejón (34) reported an association between the presence of myoma and the need for subsequent hysterectomy.

Four (1.9%) cases of hysterectomy resulted directly from treatment failure, one patient due to impaired function of the device and three others due to perforation of the uterus. One of these latter three patients required hysterectomy only four minutes after EA surgery due to the rupture of an arteriovenous malformation (AVM). Although AVM is a contraindication for EA, the 34-year-old patient desired uterine preservation and thus underwent EA therapy after giving informed consent for the hysterectomy, if required, so that the surgical team were prepared for the need for hysterectomy while undertaking the EA surgery. Rosati et al. (14) reported that of 5.2% of hysterectomies, 3.9% were directly due to treatment failure. Similarly, Comino and Torrejón (34) found that half of the 18% of hysterectomies occurring in their study were directly due to treatment failure. In our study, 95.8% cases of hysterectomy were performed in the first three years subsequent to EA, the majority within the 6 months and 12 months, which is consistent with the results of Longinotti et al. (35) study.

In this study, the most frequent adverse events were blood discharge (90.6%), vaginosis, malodorous discharge (4.3%), uterine rupture (1.4%), and extreme and prolonged suprapubic pain (0.5%). These were not unexpected given previous research (11,32,33,34). A study audit of more than 10,000 EA surgery patients from the UK found an overall complication rate of 4.4%. The most frequent complications were hemorrhage (2.4%), uterine perforation (1.5%) and cardiovascular and respiratory complications (0.5%) (36). In the Gimpelson (37) study, the only complication was uterine perforation (0.4%).

In the present study, the likelihood of a lack of treatment response and the risk of hysterectomy was higher in older patients, both of which can be related to the hormonal causes of AUB and is consistent with the literature (17,19,38). Nakamura et al. (39) showed that age was associated with recurrence of menorrhagia and re-surgery. These authors also suggested that the EA surgery may be less effective for younger women with myomas, despite the longer period of time until the onset of menopause (17,19,34,38).

The perioperative uterus size was greater than 10 cm in 85.7% of EA treatment non-responders and in 100% of perforation cases in our series. This suggests that uterine size may be an important criterion for selecting patients for EA to reduce the risk of treatment failure. Nakamura et al. (39) showed uterine cavity length (≥10 cm) was an independent risk factor for recurrence of menorrhagia and re-surgery. Larger uterine cavity length may be associated with more aggressive characteristics of myomas and thus it is not surprising that they are associated with an increased risk for recurrence and re-surgery (39). Furthermore, our series included six patients (42.85%) with myomas, endometrial polyps, and adenomyosis who proved resistant to EA treatment. We found that EA tended to be less effective in this patient population, than in women with normal, simple and complex endometrial hyperplasia. Nakamura et al. (39) showed that EA was less effective in women with myomas and adenomyosis. This study suggested that the thickened myometrium in women with adenomyosis impaired the effectiveness of EA treatment and suggested that multiple rounds of EA treatment may more successfully control menorrhagia in cases with adenomyosis.

The incidence of pregnancy after EA surgery in our study was 1.4%, in which two of three pregnancies were successful. In contrast Kohn et al. (40) reported that 85% of pregnancies following EA were terminated with abortion or due to ectopic pregnancy. This contradiction may simply be an effect of small sample size of this group in our study. It is important to make EA patients aware that EA surgery is not a contraceptive method and should apply reliable or permanent contraceptive techniques until menopause.

## Conclusion

The results of this study showed that the EA surgery with Cavaterm Thermal Balloon was an effective treatment for AUB and had satisfactory results in terms of amenorrhea and treatment response levels. In addition, the patient satisfaction rate was favorable and the procedure is safe and is associated with a very low rate of postoperative adverse events. However, our findings indicate that EA surgery may be more effective for younger patients. Also, our findings indicate EA surgery may be less effective for women with myomas, endometrial polyps, adenomyosis and a larger uterus. Further research with larger sample sizes are needed to confirm which of these clinical parameters affects the success of EA surgery in AUB and may then be used to select the most appropriate patient groups for this type of treatment.

## Figures and Tables

**Table 1:**
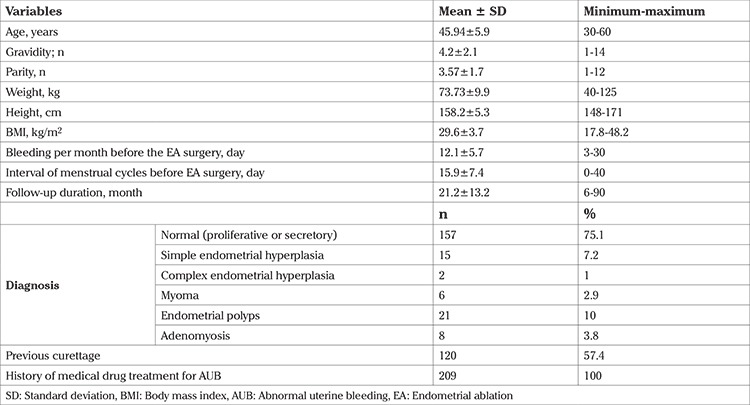
Patient characteristics of the whole cohort (n=209)

**Table 2:**
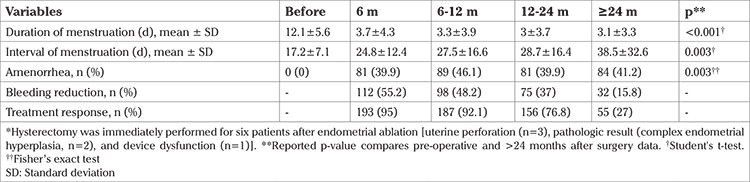
Primary outcomes in patients before and after endometrial ablation surgery (n=203*)

**Table 3:**
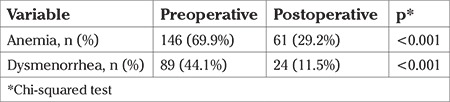
The comparison of anemia and dysmenorrhea before and six months after endometrial ablation ablation (n=209)

**Table 4:**
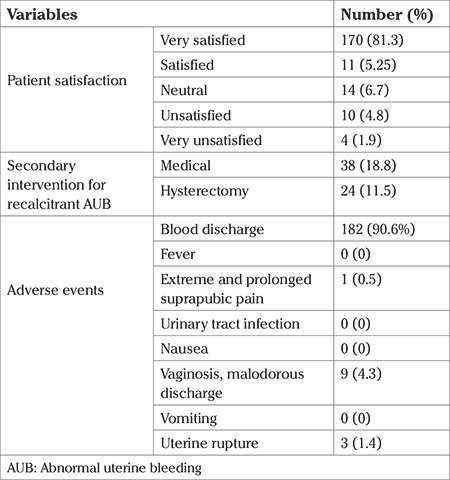
The result of patient satisfaction, secondary intervention and adverse events after endometrial ablation surgery (n=209)

**Table 5:**
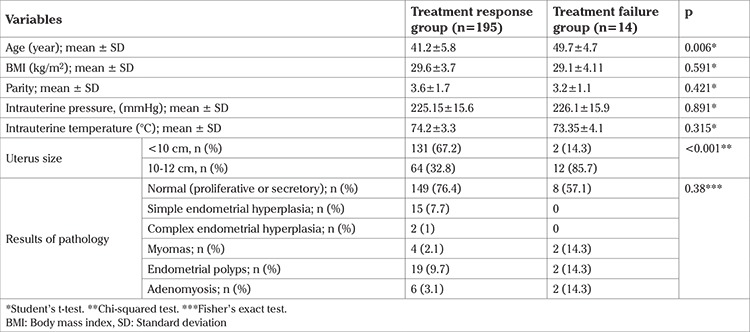
Comparison of variables between treatment responders and treatment failure groups
